# Can Neutrophil Lymphocyte Ratio Predict the Likelihood of Suicide in Patients with Major Depression?

**DOI:** 10.7759/cureus.2510

**Published:** 2018-04-19

**Authors:** Gozde Gundogdu Meydaneri, Sertac Meydaneri

**Affiliations:** 1 Psychiatry, Sancaktepe Training Hospital; 2 Orthopedics, Maltepe University Faculty of Medicine

**Keywords:** suicide, neutrophil/lymphocyte ratio, depression

## Abstract

Background

Neutrophil lymphocyte rate (NLR), platelet lymphocyte rate (PLR), and systemic immune inflammatory index (SIII) are rates obtained from hemogram parameters, and they are biomarkers used for the diagnoses of many diseases and their severity, and prediction of disease. In addition to the NLR, PLR, and SIII, platelets, plateletcrit (PCT), and platelet distribution width (PDW), which are platelet indices, are also investigated as biomarkers in many diseases. There are limited studies on the use of hemogram derivates (NLR, PLR, and SIII) in the diagnosis and severity determination of psychiatric disorders.

Objectives

In this study, we aimed to investigate the effect of biomarkers and the proportions of NLR, PLR, and SIII, which are obtained from hemogram parameters, to distinguish or predict the patients with major depression who are likely to commit suicide.

Materials and methods

In this retrospective study, the files of patients referred to an educational research emergency and psychiatric outpatient clinic between June 2017 and December 2017 were evaluated. Patients who had been referred to the emergency polyclinic because of suicide attempts and those with major depression diagnosed at a psychiatric clinic were evaluated in this study. All hemogram evaluations were performed using the Sysmex XT-2000i Automated Hematology Analyzer (GMI, MN, USA).

Results

Twenty-seven suicide patients and 26 major depression groups meeting the study acceptance criteria were included in the evaluation. Of these patients, 40 were female and 13 were male. There was no difference between the groups in terms of age, body mass index, and complete blood count (CBC) parameters such as white blood cell (WBC), neutrophil count, lymphocyte count, eosinophil count, monocyte count, platelet indices, and NLR, PLR, SIII, which were obtained from the hemogram.

Conclusion

We found that the neutrophil to lymphocyte ratio and other biomarkers obtained from hemograms were higher in patients with major depression than those who had suicide attempts, but we found that this was not statistically significant.

## Introduction

Major depression is a clinical condition that disrupts the ordinary flow of daily life and can manifest across all ages including the young and old [1]. Patients with this disorder have a predisposition to suicide and this probability is higher in patients who are left untreated [2].

Neutrophil lymphocyte rate (NLR), platelet lymphocyte rate (PLR), and systemic immune inflammatory index (SIII) are rates obtained from hemogram parameters, and they are biomarkers used for the diagnoses of many diseases and their severity, and prediction of disease [3,4]. In addition to the NLR, PLR, and SIII, platelets, mean platelet volume (MPV), plateletcrit (PCT), and platelet distribution width (PDW), which are platelet indices, are also investigated as biomarkers in many diseases [5-7]. However, the usage of hemogram derivates (NLR, PLR, and SIII) in the diagnosis and severity determination of psychiatric disorders is limited [8-10].

In this study, our purpose was to investigate the effect of biomarkers and the proportions of NLR, PLR, and SIII, which are obtained from hemogram parameters, to distinguish or predict the patients with major depression who are likely to commit suicide.

## Materials and methods

In this retrospective study, the files of patients who were referred to an educational research emergency and psychiatric outpatient clinic between June 2017 and December 2017 were evaluated. Patients who had been referred to the emergency polyclinic because of suicide attempts and those with major depression diagnosed at a psychiatric clinic were evaluated in this study. Patients under 18 years of age, patients whose CBC verification was not seen on the application, patients with psychiatric disease who additionally had systemic disease, patients with upper respiratory and other infectious diseases within the last three weeks, steroid users, and those with medication / hematologic diseases that would affect the distribution of hemogram parameters were excluded from the study. Patients using multiple antidepressants, clozapine, lithium, and electroconvulsive therapy (ECT) in the last three months were not included in the study. Patients who attempted suicide in the first four hours following drug intake were included in the study. Patients who were followed up by the psychiatric outpatient clinic and whose blood count was measured were enrolled in the study.

Patients having any hematologic-biochemical-serologic anomalies such as end-stage hyperlipidemia, hyperthyroidism, anemia, vitamin deficiency (vitamin D and B12), leukocytosis, leukopenia, and patients without adequate information in the file were excluded. The age, gender, body mass index, medication and medication usage, and hemogram data of patients were recorded.

All hemogram evaluations were performed using the Sysmex XT-2000i Automated Hematology Analyzer (GMI, MN, USA). CBC derivations were calculated using the following formulas:

NLR: Neutrophil count / Lymphocyte count

PLR: Platelet count / Lymphocyte count

SIII: (Neutrophil count x Platelet count) / Lymphocyte count

For statistical analysis, the Number Cruncher Statistical System (NCSS) 2007 and PASS (Power Analysis and Sample Size) 2008 Statistical Software (Utah, USA) programs were used. When study data were evaluated, we used descriptive statistical methods (Mean, Standard Deviation, Median, Frequency, Rate, Minimum, and Maximum), and the Mann Whitney U test was used for two groups of quantitative data with no normal distribution.

## Results

Out of the 270 patients included in our study, 89 patients were referred to the emergency polyclinic due to attempted suicide, while 181 patients with major depression underwent control examination in the psychiatry outpatient clinic.

**Figure 1:**
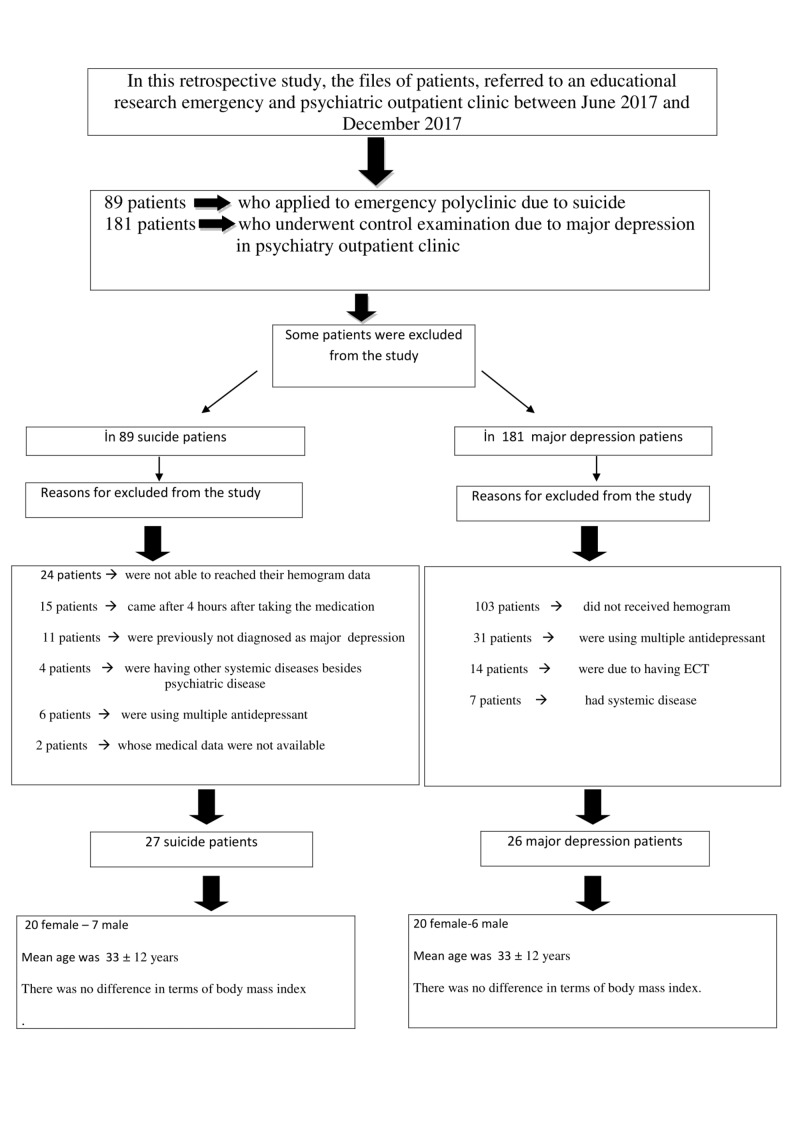
Flow chart ECT - electroconvulsive therapy

Out of the 89 patients who attempted suicide, some were excluded from the study: 24 patients who had attempted suicide were not able to obtain their hemogram data, 15 patients presented after four hours after taking the medication (drug intake or uptake of the medicine), 11 were previously not diagnosed with major depression, four had other systemic diseases besides psychiatric disease, six were using multiple antidepressants or had ECT, and for two of them medical data were not available. The flow diagram of the study is demonstrated in Figure 1.

Out of the 181 patients with major depression who were followed up by the psychiatric polyclinic, some were excluded from the study: 103 patients did not receive a hemogram, 31 were using multiple antidepressants, 14 had ECT, and seven patients had systemic diseases.

As a result, 27 suicide patients and 26 major depression patients meeting the study acceptance criteria were included in the evaluation. Out of these patients, 40 were female and 13 were male. When all patients' ages were evaluated, the average age was 33 ± 12 years and there was no difference between the groups (Table 1).

**Table 1:** Demographic data by groups

	All Patients (n: 53)	Group MD (n: 26)	Group S (n: 27)	p
Female/Male	40/13	20/26	20/27	>0.05
	Mean±SD	Mean±SD	Mean±SD	
Age	33±12	35±11	32±13	>0.05
BMI	25±7	26±5	25±6	>0.05

There was no difference in terms of body mass index either.

There was no statistically significant difference between the groups in terms of CBC parameters: WBC, neutrophil count, lymphocyte count, eosinophil count, monocyte count, platelet indices, and NLR, PLR, SIII, which were obtained from the hemogram (p> 0.05, Table 2).

**Table 2:** Distribution and evaluation of hemogram data according to groups MD: Major Depression, S: Suicide; Hb, Hemoglobin; Hct, Hematocrit; WBC, White Blood Cells; NEUT, Neutrophil; LYMHO, Lymphocytes; MCV, Mean Corpuscular Volume; RDW, Red cell Distribution Width; PLT, Platelets; PDW, Platelet Distribution Width; MPV, Mean Platelet Volume; PCT, Plateletcrit; NLR, Neutrophil lymphocyte Ratio; PLR, Platelet Lymphocyte Ratio, SII, Systemic immune-inflammatory index.

	All Patients (n: 53)		Group MD (n: 26)		Group S (n:27)		p
	Mean	SD	Mean	SD	Mean	SD	
WBC	7151,321	1613,11	6942,69	1676,34	7352,22	1554,58	0,81
NEUT	4288,113	1663,67	3897,69	1520,85	4664,07	1735,62	0,61
LYMHO	2310,189	615,82	2227,31	625,18	2390,00	607,59	0,82
Monocyte	475,8491	148,90	488,85	184,68	463,33	105,90	0,13
Eosinophils	185,8868	262,62	201,15	359,27	171,19	115,61	0,22
PLT	261	70,00	245	54	277	80	0,32
MPV	8,2	1,72	8,53	1,74	7,89	1,66	0,49
PCT	0,21	0,05	0,21	0,06	0,21	0,05	0,35
PDW	17,36245	1,16	17,04	1,26	17,67	0,97	0,71
NLR	1,946661	0,85	1,85	0,81	2,04	0,89	0,54
PLR	119,6983	40,48	118,55	42,39	120,81	39,34	0,73
SII	516642,6	295209,00	459863,21	240214,06	571319,05	335387,62	0,09

## Discussion

In this retrospective study we aimed to determine the roles of the biomarkers obtained from hemogram parameters and the hemograms to predict suicide attempt in patients with major depression; we have not reached a conclusion that biomarkers such as NLR, PLR, and SIII could be used in predicting patients who may attempt suicide. There was no significant difference between the groups.

Major depression remains an important health problem in terms of cost of treatment, and, in some cases, a significant decline in the quality of life of the patients [11]. Suicide attempt is a preventable cause of death that is common in patients with major depression, it affects the entire population, is seen higher in women, and is among the first causes of death [10,11,12]. Foreseeing patients who may make a suicide attempt, providing the necessary treatment and care, and rehabilitation are a life-saving prescription [13]. It has been reported that some biomarkers can be used to predict these patients [14].

Because the hemogram is a cheap, easily accessible diagnostic test, it is the most commonly used laboratory method. There are studies investigating the utility of the parameters obtained by the hemogram and biomarkers such as NLR, PLR, and SIII as well as the ratios of these parameters in the diagnosis of and treatment response in psychiatric diseases [8-10,14,15]. We especially noticed that patients who used clozapine were excluded from the study because it is known that clozapine would affect the function and number of neutrophils.

A study comparing hemograms of patients with major depression and normal healthy subjects reported that NLR is significantly higher in patients with major depression than in healthy control groups [10]. In another psychiatric disorder, bipolar disorder, the relationship between cognitive functions and NLR and PLR was investigated and reported as a negative correlation between NLR and cognitive function [16,17]. Another study comparing the hemogram parameters of patients with schizophrenia and bipolar disorder reported that NLR was higher in patients with schizophrenia than in patients with bipolar disorder [18].

In a study investigating the efficacy of NLR in identifying suicide risk in patients with bipolar disorder, it was reported to be a successful biomarker in predicting patients with positive family history [19]. We did not investigate the family history in our work. In another study, it was reported that in patients with major depressive disorder, the NLR was correlated with severity of illness and cardiovascular risk factors [20].

To our knowledge there are limited studies about the roles of biomarkers such as NLR and PLR in diagnosis and severity of psychiatric disorders. The study design is generally a comparison of the data of the control group and those of the psychiatric patients. Our work is distinct from similar studies in terms of methodology. In our study, patients with major depression and under treatment were compared with those with major depression and suicide. But we could not statistically distinguish between the two groups in terms of NLR, PLR, and SIII. However, this does not contradict studies reporting that NLR is high in patients with psychiatric disorders; because, in older studies, the NLR ratio in healthy subjects is 1.66-1.87 [3, 21]. In our study, this ratio was 1.85 in the MD group and 2.04 in the suicide group. We had an average of 1.94 in all patients. Although not statistically significant, NLR was higher in suicide-attempted patients. NLR is high in MD but not statistically significant, although it is higher in MD patients with suicide attempt.

Our work has some limitations. Because of the retrospective design, we may have caused the 'bias' despite our (utmost) care in our material method. In addition, smoking habit and obesity were reported to have effects on NLR [21]. Our results may be affected because we cannot evaluate smokers and obese patients themselves. But, in other studies, obesity and smoking habits were not considered in evaluations. We believe that prospective, controlled, blinded studies that will include subgroups according to drug use and MD severity, including the control group, will be able to overcome these problems and achieve more accurate and usable results.

## Conclusions

We found that the neutrophil to lymphocyte ratio and other biomarkers obtained from hemograms were higher in patients with major depression than those who made suicide attempts, but we found that this was not statistically significant. There is a need for controlled, prospective studies with family history to find out whether or not NLR can predict suicide.
